# Elevated expression of ABCB5 in ocular surface squamous neoplasia

**DOI:** 10.1038/srep20541

**Published:** 2016-02-04

**Authors:** Passara Jongkhajornpong, Takahiro Nakamura, Chie Sotozono, Maho Nagata, Tsutomu Inatomi, Shigeru Kinoshita

**Affiliations:** 1 Department of Ophthalmology, Kyoto Prefectural University of Medicine, Kyoto, Japan; 2 Department of Ophthalmology, Ramathibodi Hospital, Mahidol University, Bangkok, Thailand; 3 Department of Frontier Medical Science and Technology for Ophthalmology, Kyoto Prefectural University of Medicine, Kyoto, Japan

## Abstract

ATP-binding cassette subfamily B member 5 (ABCB5) is a new member of the ATP-binding cassette superfamily and has been reported as a novel marker for limbal stem cell (LSC), which is essential for corneal homeostasis. ABCB5 expression has also been discovered in the subpopulation of several cancer cells containing the cancer stem cell (CSC). However, the pathogenetic relationship between LSC and CSC and ABCB5 in the ocular surface squamous neoplasm (OSSN) is still entirely unknown. To improve understanding of the role of ABCB5 in OSSN, we performed immunohistochemistry for ABCB5 in nine OSSN case series. While expression of ABCB5 is restricted to the basal epithelial cell layer in the normal limbus, elevated expressions of ABCB5 were clearly observed in all OSSN, and there was some breadth in the range of intensity of ABCB5 expression. Interestingly, the elevated expression patterns of ABCB5 in OSSN could be classified in three categories: perivascular, marginal and diffuse patterns. Our findings demonstrated for the first time that the expression of ABCB5 was upregulated in OSSN and that elevated expression of ABCB5 may be involved in the pathogenesis of OSSN.

Ocular surface squamous neoplasia (OSSN) is one of the most common tumors of the cornea and conjunctiva^1^,^2^,^3^. Lee *et al*. proposed the term “OSSN” in 1995 with reference to the disease spectrum comprising corneal intraepithelial neoplasia (CIN) (subdivided into mild, moderate and severe dysplasia), carcinoma *in situ* (CIS) and invasive squamous cell carcinoma (SCC)^1^. The incidence of OSSN varies with geographical region, depending on racial characteristics and latitude^4^. Although surgical en bloc excision with concomitant double freeze-thaw cryotherapy and adjuvant topical chemotherapy has been the standard treatment for OSSN^2^,^5^, the recurrence rate was as high as 20% at 5-year follow-up^6^,^7^. OSSN occurs predominantly in the limbal area^3^,^8^ where the limbal stem cell (LSC) niche resides, but the molecular mechanism and pathogenesis of OSSN are still unknown.

ATP-binding cassette subfamily B member 5 (ABCB5) is a new member of the ATP-binding cassette superfamily and has been identified as an important factor in regulating progenitor cell fusion, multidrug sensitivity and cellular melanogenesis^9^,^10^. Expression of ABCB5 has also been discovered in the subpopulation of several cancer cells that contain the cancer stem cell (CSC)^11^,^12^,^13^,^14^,^15^,^16^. Most recently, ABCB5 has been identified as a novel marker for LSC and is known to be responsible for stem cell differentiation and stem cell immortality, as well as for anti-apoptotic activity^17^. However, the pathogenetic relationship between LSC and CSC and ABCB5 in OSSN remains entirely unknown.

In the present study, to gain some insight into the possible function of ABCB5 in tumor progression in OSSN, we performed immunofluorescence analysis that clearly revealed the elevated expressions of ABCB5 in all OSSN cases. These findings demonstrated conclusively for the first time that the expression of ABCB5 is upregulated in OSSN and that elevated expression of ABCB5 may be involved in the pathogenesis of OSSN.

## Results

### Clinical presentations

**Figure 1:**
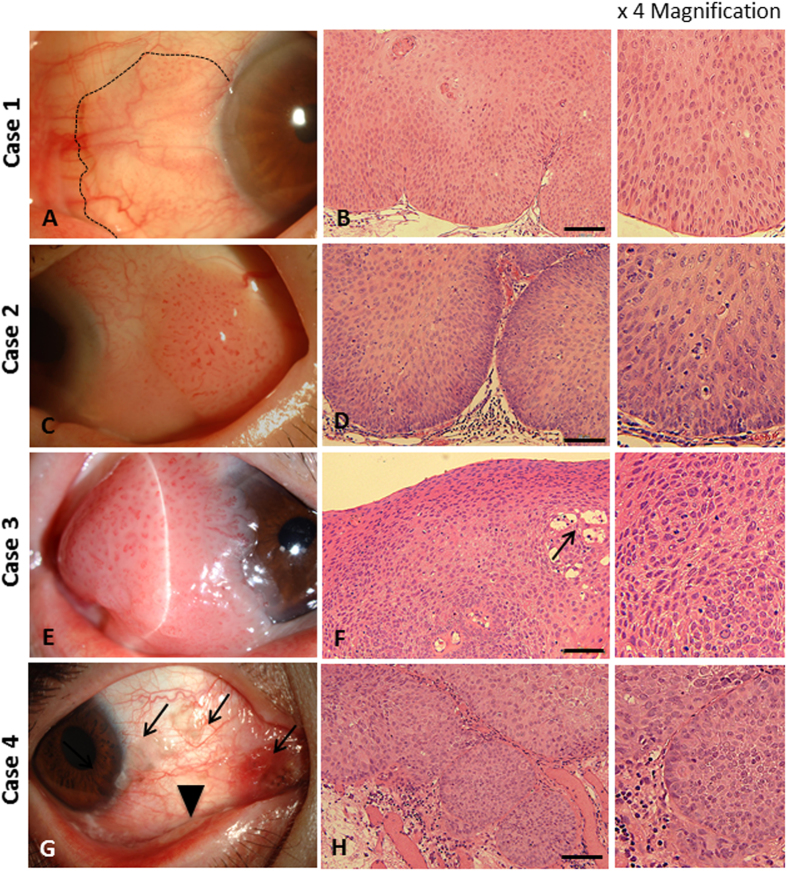
Clinical presentation and histopathology of CIN and CIS. Case 1 (CIN): Elevated mass with frond-liked vascular projection located at nasal limbus (dash line) (**A**); dysplastic change involving almost full thickness of epithelial layer with intact basement membrane (**B**). Case 2 (CIS): Papilliform mass with typical hairpin vascular configuration located at temporal bulbar conjunctiva (**C**); dysplastic cells extending to all of the epithelial layers, with intact basement membrane (**D**). Case 3 (CIS): Large papilliform mass located at nasal limbus with adjacent corneal invasion (**E**); full thickness of epithelium showing dysplastic configuration with multiple neoplastic vascularization (arrow), corresponding with clinical findings (**F**). Case 4 (CIS): Multifocal nodular masses located at nasal bulbar conjunctiva; some pigmentation (arrows) and conjuctival fibrosis (arrow head) were observed (**G**); multiple dysplastic epithelial lobules were found, with no basement membrane invasion (H). Scale bars = 100 μm.

**Figure 2:**
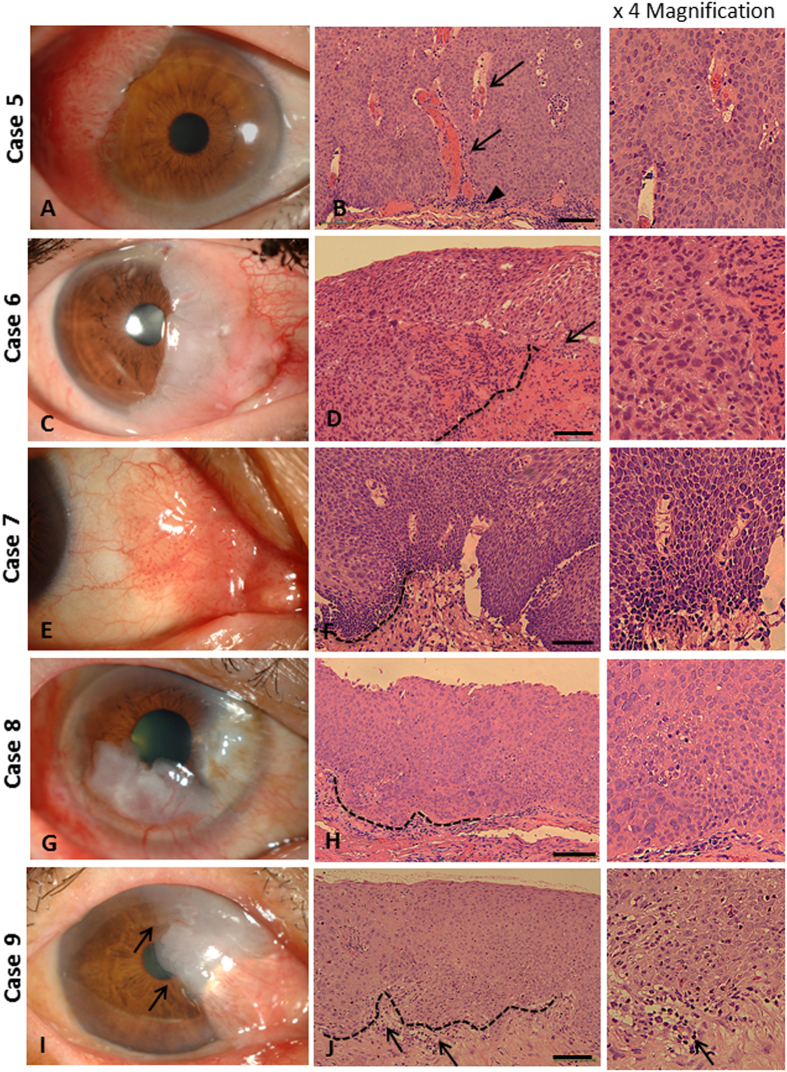
Clinical presentation and histopathology of SCC. Case 5: Large papilliform mass with perforating vascular tufts occurring at the superotemporal limbus, with adjacent corneal invasion (**A**); neoplastic cells were observed in full thickness of epithelial layer, invading stromal connective tissue with high neovascularization (arrow); some inflammatory cells were observed along the tumor margin (arrow head) (**B**). Case 6: Opaque greyish gelatinous mass with fimbriated margin occupying the nasal limbus, with corneal involvement (**C**); pleomorphic cells invaded the subepithelial connective tissue (dash line), with some inflammatory reaction (arrow) (**D**). Case 7: Papilliform mass located at nasal bulbar conjunctiva and caruncle (**G**); pleomorphic cells with dense large nucleus and basophilic cytoplasm, packed and exophytically spreading into the connective tissue (dash line) (**F**). Case 8: Leukoplakic mass involving two-thirds of the corneal limbus (**G**); pleomorphic cells were observed in all epithelial layers, extending into the subepithelial stroma (dash line) (**H**). Case 9: Gelatinous mass with fimbriated leading margin (arrow), connected to pterygium, appearing at nasal limbus (**I**); pleomorphic cells invaded basement membrane (dash line) with inflammatory reaction (arrows) (**J**). Scale bars = 100 μm.

Nine OSSN patients participated in the study, including 4 males (4/9 patients, 44.44%) and 5 females (5/9 patients, 55.56%) with a mean age of 68 years (range 40–87 years) (Table 1). Six of nine cases (66.67%) involved the right eye, with lesions located at the nasal side in seven of nine eyes (77.78%). Based on clinical appearance, the 9 OSSNs were classified into 4 groups, including 5 eyes of papilliform type (Figs 1A,C,E and 2A,E); 2 eyes of gelatinous type (Fig. 2C,I); 1 eye of leukoplakic type (Fig. 2G) and one other eye (unclassified) (Fig. 1G). All patients received en bloc excision with application of mitomycin C (MMC). Cryotherapy was applied in one case (case 7). Amnion transplantation (AMT), limbal transplantation (LT) or keratoepithelioplasty (KEP) was performed on the basis of a case-based evaluation (Table 1). Post-operative chemotherapy with 5-fluorouracil (5-FU) was prescribed for 5 patients (cases 4–8). For 7 patients of more than 2-year follow-up, local recurrence was observed in 2 eyes (28.6%, one CIS and one SCC). Recurrence was detected in one patient following 2 cycles of post-operative 5-FU administration and was successfully treated with re-excision. Human papilloma virus 16 (HPV 16) was detected in one patient (case 7). From our clinical observations, CIS, CIN and SCC are generally similar in appearance, making it difficult to distinguish between them in a clinical setting. Clinical presentations are summarized in Table 1.

### Histopathology

Based on histopathological features, the OSSNs were classified as 1 eye with CIN, 3 eyes with CIS and 5 eyes with SCC. CIN and CIS (Fig. 1B,D,F,H) showed intraepithelial dysplastic change originating from the basal epithelial layer, with intact basement membrane. Dysplastic cells were characterized by bizarre hyperchromatism, high nucleus to cytoplasm ratio and the presence of atypical mitotic figures. In case 1, almost the full thickness of the epithelial layer was occupied by dysplastic cells consistent with the diagnosis of CIN (severe dysplasia) (Fig. 1B). Full thickness dysplastic change was observed without basement membrane invasion in cases 2–4, indicating CIS (Fig. 1D,F,H). In case 3, multiple fronds of neovascularization were observed, correlated with the clinical appearance of a fine hairpin vascular pattern (Fig. 1E,F).

For SCC (cases 5–9), pleomorphic cells were observed with dense cytoplasm, coarse chromatin and high nuclear to cytoplasmic ratio (Fig. 2B,D,F,H,J). The neoplastic cells invaded the basement membrane and reached the subepithelial connective tissue. Loss of cellular polarity was a consistent characteristic of all cases of SCC. Multiple perforating vessels, observed in cases 5 and 7, conformed to the clinical appearance of the papilliform type (Fig. 2A,B,E,F, respectively). The inflammatory response was usually detected at the connective tissue adjacent to the tumor margin (Fig. 2B,H,J). Although the clinical presentation of CIS, CIN and SCC was similar in appearance, the pathological findings clearly differed.

### Immunohistochemistry

**Figure 3:**
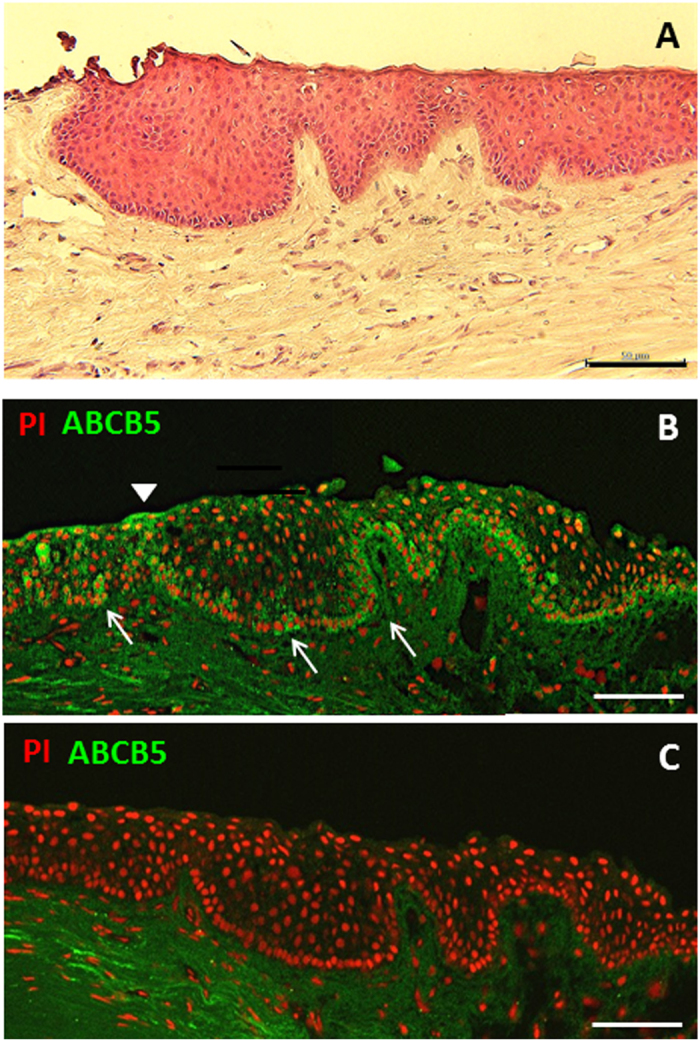
Histopathology and ABCB5 expression pattern of normal limbus. Well stratified epithelial cells with finger-like projection into stromal connective tissue at the limbus (**A**); ABCB5 expressed clearly at the basal epithelial layer of the limbus (arrows) (**B**) although no expression was observed in the suprabasal layers (**B**); isotype control showed no expression of ABCB5 in the epithelial layer (**C**). Scale bar = 100 μm.

In normal limbal tissues, we observed a well-organized stratified epithelium with intact cellular polarity (Fig. 3A). The palisade of Vogt structure was clearly identified by the finger-like figure extending into the subepithelial connective tissue. In the normal limbus, ABCB5-positive cells were confined to the basal epithelial layer and were hardly observed in the suprabasal epithelial layer (Fig. 3B). An isotype-matched control showed negative expression of ABCB5 in the epithelial layer, with some background staining at the subepithelial connective tissue (Fig. 3C).

**Figure 4:**
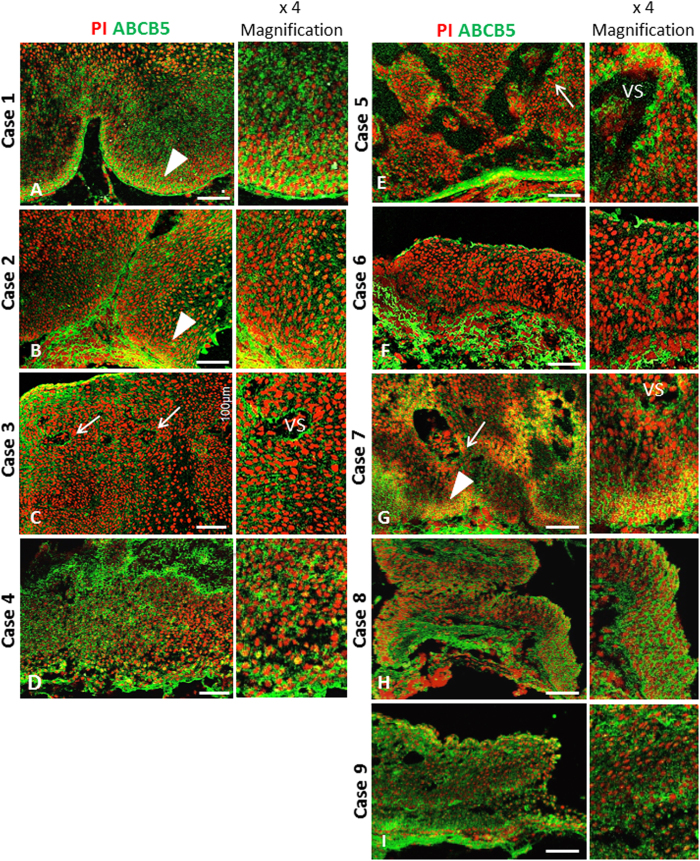
Expression patterns of ABCB5 in OSSN. Four OSSNs (CIN and CIS, cases 1–4) and 5 OSSNs (SCC, cases 5–9) showed aberrant ABCB5 expression as compared to normal limbus. Three specific patterns were observed: *perivascular pattern*, identified by high expression of ABCB5 around vascular structures (arrow) in cases 3 (**C**), 5 (**E**) and 7 (**G**) and *marginal pattern*, showing high ABCB5 expression at the basal tumor margin (arrow heads) in cases 1 (**A**), 2 (**B**) and 7 (**G**). Other cases showed diffuse pattern of ABCB5 overexpression (**D,F,H,I**). VS: vascular structures. Scale bar = 100 μm.

In all 9 OSSNs, ABCB5 expression patterns appeared different from those found in normal limbus, most of them showing significant ABCB5 overexpression as compared to normal limbus (Fig. 4). Moreover, the pattern of ABCB5 expression in OSSN was not confined to the basal epithelial layer but also appeared at the suprabasal and superficial cells. Three specific patterns were observed: 1) a perivascular pattern, in which high expression of ABCB5 was observed in a particular area surrounding the vascular structures in cases 3, 5 and 7 (Fig. 4C,E,G, respectively); 2) a marginal pattern, in which high ABCB5 expression was observed at the basal margin of tumors in cases 1, 2 and 7 (Fig. 4A,B,G, respectively) and 3) a diffuse pattern, in which ABCB5 was diffusely expressed in all epithelial layers in cases 4, 6, 8 and 9 (Fig. 4D,F,H,I). The intensity and pattern of ABCB5 expression differed significantly from normal limbus, but we could find no significant difference in ABCB5 expression pattern among CIN, CIS and SCC.

## Discussion

Based on the immunohistochemical studies, restricted expression of ABCB5 was found in normal limbal epithelium at the basal epithelial layer, aligning with the recent study by Ksander *et al*.^17^. It was known that OSSN develops predominantly in the limbal region and is histologically composed of the group of dysplastic cells originating from the basal epithelial layer, suggesting the possibility of transdifferentiation from limbal stem cell. However, this concept has not been experimentally proved. Within the limitations of small samples, the present study demonstrated the particular subpopulation in OSSN, using the immunohistochemical study of ABCB5. Interestingly, this aberrant pattern of ABCB5 expression was observed in all OSSNs. Although three specific patterns were detected, ABCB5 expression in OSSN presented most often as a non-specific pattern through the whole thickness of the tumor (Fig. 4). We could find no relationship between patterns of expression and tumor progression. Further tumor growth studies using *in vitro* colony-forming assay and *in vivo* xenotransplant experiments will be required to identify cancer stem cells or the cell lineage of OSSN.

Grimm *et al*. have demonstrated that ABCB5 is significantly overexpressed in human oral squamous cell carcinoma (OSCC) as compared to normal oral squamous epithelium, which is confined to the basal cell layer^18^. In addition, ABCB5 expression was also associated with tumor progression and survival rate in cases of OSCC^18^. As for OSCC, ABCB5 overexpression was also found in OSSN. Given the limited sample and the various expression patterns of ABCB5 observed in this study, the relationship between ABCB5 expression and tumor progression could not be properly evaluated. Additional studies, including a greater number of clinical cases, are therefore needed to clarify this relationship.

During cancer therapy, ABCB5 has been demonstrated to be involved in chemoresistant stem cell, as well as drug efflux transportation of doxorubicin in human melanoma^12^,^19^,^20^ and chemoresistance to 5-Fluorouracil (5-FU) in colorectal cancer^21^. Mitomycin C, 5-FU and interferon are currently considered to be the main options for treating OSSN. Recurrence following 5-FU therapy has rarely been reported, and re-administration of the same agent remains effective for treatment in cases of recurrence^22^,^23^. In our series, there were 2 OSSNs with tumor recurrence; one received postoperative adjuvant 5-FU while the other did not. Both were successfully managed by means of repeated surgical en bloc excision. The interesting questions are whether ABCB5 determines drug efflux in OSSN and why multidrug resistance is rarely found in cases of OSSN where ABCB5 is highly expressed. We hypothesized that ABCB5 might not be involved in the chemoresistance mechanism of tumor cells in OSSN, but more work is needed to conclusively establish this.

Taken together, the clinical findings, histopathology and immunohistochemical study demonstrated first of all that the elevated ABCB5 expression pattern in all OSSNs suggests up-regulation and misarrangement of specific subpopulations containing some undifferentiated stem cell-like phenotype. Our findings provide an initial basis for further exploration of the particular subpopulation in relation to the pathogenesis and advanced treatment of OSSN.

## Materials and Methods

### Subjects

Nine pathological specimens were obtained from OSSN patients who underwent surgery at Kyoto Prefectural Hospital University of Medicine (KPUM). The research protocol was approved by the institutional review boards (IRBs) for Human Studies of Kyoto Prefectural University of Medicine, and prior informed consent was obtained from all patients in accordance with the tenets set forth in the Declaration of Helsinki for research involving human subjects. Demographic (age, gender and laterality), clinical presentation, treatment and follow-up data were reviewed. Anterior segment photographs were collected and analyzed by two cornea specialists (C.Z. and T.I.). From histopathology, the OSSNs were classified into three groups (CIS, CIS and SCC). CIN was diagnosed by intraepithelial dysplastic changes originating from the basal layers of the conjunctival or corneal epithelium, and CIS was diagnosed where atypical cells were found in the full thickness of the epithelial layer with no basement membrane invasion. SCC was defined by anaplastic change of stratified squamous epithelial cells extending into the subepithelial connective tissue. For normal control, three corneoscleral rims were obtained from US corneal donors (SightLife Eye Bank, USA).

### Immunohistochemistry (IHC)

Immunohistochemical studies were performed according to our previously described method^24^,^25^. Briefly, pathological specimens were immediately embedded in OCT compound and stored at −80 °C following the operation. Eight μm frozen sections were placed on silanized slides (Dako, Glostrup, Denmark) and allowed to dry before fixing with 100% acetone at 4 °C for 10 minutes. After washing with phosphate-buffered saline (PBS) and 0.15% TRITON X-100 (Dow Chemical Company, MI, USA) for 10 minutes each, slides were subsequently blocked with 2% bovine serum albumin (BSA; Sigma-Aldrich Co. LLC., MO, USA), diluted by goat serum (Jackson ImmunoResearch Laboratories, Inc., PA, USA) at room temperature (RT) for 1 hour to avoid nonspecific antibodies. The sections were then incubated with primary antibodies (Rabbit anti-human ABCB5 polyclonal antibody NBP1-50547 × 500; Novus Biologicals, CO, USA) at RT for 30 minutes and washed with 0.15% TRITON X-100 for 10 minutes (x 2), followed by PBS for 10 minutes. Isotype control at the same concentration as the primary antibodies was performed to exclude non-specific staining. Appropriated secondary antibodies (Goat anti-rabbit 488; MP Biomedicals, CA, USA) were incubated for 30 minutes. After repeat washing (as in the primary antibody process), all slides were mounted in propidium iodide (PI; Vectashield; Vector Laboratories Inc., CA, USA) and then examined under a confocal microscope (Fluoview FV1000; Olympus Co., Tokyo, Japan).

## Additional Information

**How to cite this article**: Jongkhajornpong, P. *et al*. Elevated expression of ABCB5 in ocular surface squamous neoplasia. *Sci. Rep.* **6**, 20541; doi: 10.1038/srep20541 (2016).

## Figures and Tables

**Table 1:** Demographics, clinicopathological features, treatments and recurrence of 9 OSSNs.

Case No.	Pathological Diagnosis	Age	Gender	Laterality	Clinical appearance	Surgical Treatment	Adjuvant Chemotherapy	F/U	Recurrence	PCR for HPV16, 18
1	CIN	76	Female	L	Papilliform	En-block excision + MMC + KEP + AMT	–	2 years	No	Negative
2	CIS	81	Female	L	Papilliform	En-block excision + MMC + KEP + AMT	–	3 years	No	Negative
3	CIS	87	Female	L	Papilliform	En-block resection + MMC + AMT	–	5 years	No	NA
4	CIS	40	Female	R	Atypical	En-block excision + MMC+ AMT	5-FU × 3 cycles	2 years	No	Negative
5	SCC	57	Male	R	Papilliform	En-block excision + MMC + AMT	5-FU × 1 cycles	6 months	No	Negative
6	SCC	52	Female	R	Gelatinous	En-block excision + MMC + KEP + AMT	5-FU × 2 cycles	4 months	No	NA
7	SCC	61	Male	R	Papilliform	En-block excision + MMC + CRYO + AMT	5-FU × 4 cycles	7 years	No	Positive HPV 16
8	SCC	80	Male	R	Leukoplakic	En-block excision + MMC + KEP + AMT	5-FU × 2 cycles	7 years	No	Negative
9	SCC	77	Male	R	Gelatinous	En-block excision + MMC	–	2 years	Yes	Negative

AMT=Amnion transplantation, CIN=Conjunctival intraepithelial neplasia, CIS= Carcinoma *In situ,* CRYO=Cryotherapy, HPV=Human papilloma virus, KEP=Keratoepitheliotoplasty, MMC=Mitomycin C, NA=Not available, PCR=Polymerase chain reaction, 5-FU=5 Fluorouracil.
